# Primary and middle school students' views on inclusive physical education: Perceptions, practices, and future directions

**DOI:** 10.1016/j.heliyon.2024.e41232

**Published:** 2024-12-14

**Authors:** Gabriele Russo, Alice Masini, Laura Dallolio, Andrea Ceciliani

**Affiliations:** a Department of Economics, Engineering, Society, and Business Organization, University of Tuscia, Viterbo, (VT), Italy; b Department for Life Quality Studies, University of Bologna, Rimini, (RN), Italy; c Department of Translational Medicine, University of Eastern Piedmont, Novara, (NO), Italy; d Department of Biomedical and Neuromotor Sciences, University of Bologna, Bologna, (BO), Italy

**Keywords:** Inclusion, Well-being, Physical activity, Teacher behavior, Exploratory factor analysis, Disabilities

## Abstract

**Background:**

Physical Education (PE) classes are vital for nurturing students' social development and promoting collaboration. This study examined how primary and middle school students perceived PE classes, with a focus on collaboration, well-being and enjoyment, teacher behavior, the inclusion of nondisabled and disabled students, and general class behavior.

**Methods:**

One-hundred and seventy students (24 primary school and 146 middle school students) were surveyed using a questionnaire.

**Results:**

Exploratory Factor Analysis identified seven factors including: well-being, activities for all students, teacher-oriented behavior, collaboration, and attention to students with disabilities. Linear regressions on each identified Factor revealed higher well-being (Factor 1) between primary and middle school students. In addition, the perceived well-being in PE classes and staying together with classmates among younger middle school students was found to be higher than that reported by their older counterparts in middle school. The analysis also revealed that males perceived a higher well-being in PE classes than females.

**Conclusion:**

The research underscores that primary and especially middle school PE teachers should promote the well-being of students by creating inclusive and enjoyable PE classes. The PE classes should consider the differences between sexes and the individual differences. Finally, this research lays the foundation for future investigations to refine inclusive PE strategies and improve students' well-being.

## 1 Introduction

Physical Education (PE) classes play a crucial role in fostering various social aspects of students such as teamwork, social inclusion, and communication [1,2]. These classes offer opportunities for students to collaborate toward common goals, such as completing team-based activities [3,4], and the interactive environment in PE makes learning enjoyable for both students and teachers. Simultaneously, it is essential that PE lessons are tailored to cater to the diverse needs of students, including those with varying skill levels and special needs.

Aligned with the Self-Determination Theory, deriving enjoyment from the process is integral to fostering intrinsic motivation for physical activities and the adoption of healthy behaviors [5,6]. In other words, people are most motivated to engage in an activity when they find it is inherently enjoyable and aligns with their personal values. Unfortunately, there are instances where PE teachers or teachers responsible for PE classes may lack the necessary competence to program enjoyable and functional physical activities, which can result in not contributing effectively to the overall development of students [7,8]. This challenge is particularly significant for disabled children, who may face additional barriers to accessing inclusive PE [9], in which, students with disabilities often find themselves in the position of spectators as their peers engage in motor activities, and in most severe cases, they are even isolated from the rest of the class (e.g.,8). For instance, Coates and Vickerman [10] underscored that students with disabilities frequently face exclusion from PE classes due to various issues. These include the determination of what disabled students are able to do [11], inadequate teacher expertise, structural barriers like a lack of training in adapted physical activity, concerns about student safety, time limitations, and large class sizes (see e.g., 7,11). Therefore, in the last two decades, the development of enjoyable and inclusive PE lessons has been rewarding for all teachers and educators[[12], [13], [14]], and the development of enjoyable and inclusive PE lessons can be beneficial for both children with and without disabilities [5,15,16].

Research indicates that children without disabilities can increase their social skills (e.g., empathy, moral and ethical principles, and reduced fear of human differences) and the perception of disabilities (e.g., self-concept and self-perception of the body [[15], [16], [17]]), while children with disabilities can enhance their psychological (e.g., inhibitory control [18]), psychosocial skills (e.g., self-perception and self-efficacy [19]), and physiological parameters (e.g., cardiovascular capacity [20,21]). However, these improvements are also associated with the type of disability. For instance, Barkley's Theoretical Model [22] highlights that attention deficit hyperactivity disorder is characterized by weak inhibition control, and PA can be a useful tool for addressing this [23,24]. From psychological, psychosocial, and physiological perspectives, it is evident that the development of inclusive, collaborative, and enjoyable PE lessons is crucial for teachers and educators aiming to enhance students' well-being.

These principles underpin the Social Ecological Model, which acknowledges that individual health outcomes are shaped by a combination of factors across multiple levels, including the individual (personal beliefs and behaviors), interpersonal (relationship and social networks), institutional (organizational practices and policies), and societal levels (broader social and cultural norms). Consequently, gaining insight into students’ perceptions within PE classes is crucial for enhancing the overall quality of educational processes.

Despite this, Wilhelmsen and Sørensen's review [25] reveals notable disparity in research focus, with fewer studies exploring students' perceptions compared to those centered on teachers' and parents' viewpoints. Therefore, this study, through a questionnaire, aimed to explore the students' experience of PE classes in terms of collaboration, well-being, teachers' behavior, the inclusion of nondisabled and disabled students, and the overall behavior of the class in primary and middle schools.

We hypothesized differences between primary and middle school were expected, as it is commonly acknowledged that primary school tends to be more inclusive than middle school. In addition, the primary school setting is often characterized by a greater degree of collaboration compared to the middle school environment [26]. The outcomes will be adjusted for participants' class (age) and sex, recognizing their potential mediation in students' perceptions [4,27]. In particular, sex differences were expected, with the common observation of greater male engagement in PE compared to females, especially in middle school. Within middle school, variations were also predicted, postulating that younger students might display increased collaboration, potentially shaping differences in their experiences.

## 2 Methods

### 2.1 Ethics and consent

Parents of children gave written informed consent and signed the privacy form after a study briefing. In addition, for students aged 12 and above, a simplified version of the informed consent and privacy form was delivered to obtain the written informed consent. The study adhered to the Helsinki Declaration and received approval from the local bioethics committee of the University of Bologna (N. Prot: 0387983).

### 2.2 Participants

One-hundred and seventy (83 females; age range: 8–14 years old) primary and middle school students completed the survey. Twenty-four (9 females) belonged to the primary schools, and 144 students (74 females) belonged to the middle school (see Table 1). The schools belonged to a multicultural Italian metropolitan city, and they were selected based on the willingness to participate and the amount of PE lessons performed during the week according to the national law (i.e., 2 h/week).

**Table 1:** Shows the sample numerosity according to type of school, class, and teacher.

l	Teacher	Class	Numerosity	Total
Primary	Teacher 1	II	16	24
	Teacher 2		8	
Middle	Teacher 3	I	44	67
	Teacher 4		23	
	Teacher 3	II	35	37
	Teacher 4		2	
	Teacher 3	III	23	42
	Teacher 4		19	

### 2.3 Instrumentation

We created a survey that analyzed several aspects of the student perception of PE classes. Specifically, we focused our attention on general well-being and enjoyment (e.g., items 5 and 6 [see Table 2]), inclusion (e.g., items 8 and 9 [see Table 2]), teachers’ behavior (e.g., item 22 [see Table 2]). The first and last authors created the questionnaire, and each item was discussed with the other authors. The survey was designed to be completed online through the Microsoft Forms platform. The initial question pool encompassed 31 items (see Table 2), and each participant responded on a Likert-5 scale from "not at all” to "a lot."

**Table 2:** Shows all questionnaire items.

Item	Question	Factor	Item	Question	Factor
1	All students are involved in Physical Education lessons	2	16	The teacher willingly works with the whole class	7
2	When someone is in difficulty, they can rely on the help of their classmates		17	I enjoy activities with students with disabilities	5
3	During the physical education class, the teacher is attentive to students with difficulties	7	18	I feel sorry for students with disabilities	
4	When someone is in difficulty, they can count on the help of the Physical Education teacher		19	Games suitable for students with disabilities are boring	5
5	I willingly participate in Physical Education classes	1	20	During Physical Education classes, we also work in pairs	
6	I have fun during Physical Education classes	1	21	During Physical Education classes, we also work in small groups	
7	During the Physical Education class, games and activities involve everyone	2	22	If someone is in difficulty, the teacher helps them	5
8	Games and activities are accessible to everyone	3	23	During Physical Education classes, we feel good together	1
9	The games are achievable by everyone	3	24	The teacher is attentive to the effort	5
10	Students with disabilities are treated well	7	25	The teacher is attentive to the result	
11	Students with disabilities are facilitated too much		26	The teacher always praises those with the best results in activities	4
12	Games and activities involve everyone	2	27	The teacher praises the most skilled	4
13	During Physical Education classes, we work a lot together	3	28	The teacher is attentive to those with worse results	
14	The teacher proposes activities suitable for the most skilled	6	29	The teacher only addresses the most skilled	6
15	The teacher only rewards the best	6	30	During Physical Education classes, I feel good	1
			31	During Physical Education hours, I feel at ease	1

### 2.4 Procedure

Teachers distributed the questionnaire link to parents or legal guardians of students under 12 and directly to students aged 12 and above. Students completed the questionnaire either during school hours or at home under the supervision of parents/legal guardians. Teachers and parents/legal guardians were instructed not to interfere with the questionnaire filling to avoid possible bias.

### 2.5 Data analysis

Data analysis was performed with R Software (v. 4.3.1www.rsoftware.com) using the RStudio framework (v. 2023.09.1, www.posit.co).

***Exploratory Factor Analysis (EFA).*** EFA was employed to identify the underlying structure of the data and to determine the number of latent factors. EFA is particularly appropriate when the aim is to explore the possible underlying and validate the factor structure of a set of observed variables without imposing a preconceived structure on the outcome. Before conducting EFA, correlation among survey items (Table S1) was calculated to analyze the interdependence among the variables (r > .80).Internal consistency and reliability of the survey and its subscales were assessed using Cronbach's α ("*ltm*” package [28]).

EFA [psych and EFA tool packages [29,30] was performed to identify the possible latent variables of the questionnaire and determine their suitability for explain our aims. The feasibility of EFA was analyzed by checking the correlation matrix (see Table S1), computing the Kaiser-Meyer-Olkin (KMO) measure of sampling adequacy, and computing the Bartlett sphericity test. Each EFA was conducted using a Promax rotation. For each Factor, we summed the points scored by each participant for each related question.

Linear regressions for parametric and non-parametric data were performed using the "*lm*” function and the "*art*” function (ARTool package [31,32]), respectively. Dependent variables, identified by EFA, were analyzed against independent variables "school” (primary *vs.* middle school) and "sex” (females *vs.* males). In addition, each school type was separately analyzed: in primary school, the independent variable was "sex,” in middle school, the independent variables were "sex” and "class.” The "teacher” confounding factor was analyzed as a confounding factor in each parametric and nonparametric linear regression analysis. Tukey post-hoc analyses, performed using the "*emmeans”* package [33], located significant differences.

## 3 Results

***EFA*.** EFA was conducted on the entire sample (170 students). The first step evaluated correlations among items; none were more than .80 (Table S1). Thus, the first EFA included all items. Cronbach's α was .78, and the KMO analysis revealed that the Measure of Sampling Adequacy (MSA) was middling (MSA = .75). However, the MSA of item 20 was lower than .50. Consequently, we decided to remove from the EFA 1.

Bartlett's sphericity test on 30 items rejected the null hypothesis (*Χ*^2^_(435)_ = 1688.33, p < .001), indicating the suitability of data for factor analysis. Cronbach's α was .79, and KMO was middling (MSA = .76). The Eigenvalues based on Squared Multiple Correlation (SMC) and the Parallel Analysis suggested considering seven factors. Therefore, we conducted an EFA considering seven factors. The analysis revealed that seven factors were sufficient to explain all items of the questionnaire (*Χ*^2^_(246)_ = 270.16, p > .05). However, items 11, 21, and 28 remained outside the EFA 1 or had a value below .40.

Cronbach's α on 25 items was .83, the KMO was middling (MSA = .80), and Bartlett's sphericity test was significant (*Χ*^2^_(300)_ = 1541.03, p < .001). The Eigenvalues and the Parallel analyses suggested that the number of factors to consider was between 4 and 7. We decided to consider 7 factors, and the EFA 3 revealed that they were sufficient to explain all items (*Χ*^2^_(146)_ = 162.43, p > .05). However, item 2 remained outside of the EFA and was removed from EFA 3.

Cronbach's α on 24 items was .81, the KMO was middling (MSA = .79), and Bartlett's sphericity test was significant (*X*^2^_(276)_ = 1413.09, p < .05). The Eigenvalues and the Parallel analyses suggested to consider seven factors. The EFA 3 revealed that 7 factors were sufficient to explain all remaining items of the questionnaire (*Χ*^2^_(129)_ = 139.84, p > .05). However, item 4 remained outside the EFA 3.

**Fig. 1:**
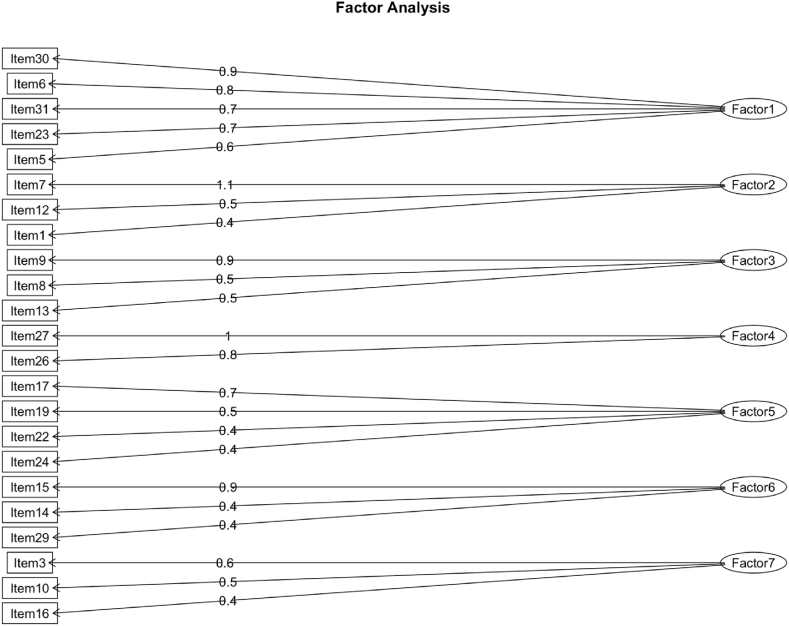
Shows the EFA 4 with the seven factors.

Cronbach's α on 23 items was .81, the KMO was middling (MSA = .78), and Bartlett's sphericity test was significant (*Χ*^2^_(253)_ = 1339.64, p < .05). The Eigenvalues and the Parallel analyses suggested to consider seven factors. The EFA 4 revealed that seven factors were, again, sufficient to explain all remaining items of the questionnaire (*Χ*^2^_(113)_ = 124.49, p > .05). The EFA 4 (see Fig. 1) identified seven factors: Factor 1 was related to the well-being of students during physical education lessons (items 30, 6, 31, 23, and 5, Cronbach's α = .84); Factor 2 encompasses items 7, 12, and 1, which were related to the physical activities designed for all students (Cronbach's α = .41); Factor 3 was related to the difficulty/plainness of the activity proposed by the teacher (items 9, 8, and 13; Cronbach's α = .66); Factor 4 was related to the oriented behavior of the teacher to the result and encompasses items 27 and 26 (Cronbach's α = .81); Factor 5 was associated with the perception of activities with students with disabilities and to collaboration among students (items 17, 19, 22, 24; Cronbach's α = .61); Factor 6 identified items related to physical activities orientation (items 15, 14, and 29; Cronbach's α = .57); and Factor 7 was related to students' attention towards nondisabled and disabled students (items 3, 10, and 16; Cronbach's α = .58).

***Primary vs. Middle schools.*** Artool analysis revealed on Factor 1 significance for the single factor "school” (F_(1,166)_ = 4.17, p = .04, η_P_^2^ = .02). Primary school students reported higher well-being than middle school students (see Table 3). The single factor “sex” was significant (F_(1,166)_ = 6.30, p = .01, η_P_^2^ = .04). The interaction “school” x “sex” was also significant (F_(1,166)_ = 5.36, p = .02, η_P_^2^ = .3, see Table 3). Post-hoc analysis revealed higher scores for the primary school females compared to the middle school females (t_(166)_ = 2.61, p = .049, d = .92) and higher scores for middle school males compared to their counterpart females (t_(166)_ = 4.15, p = .0003, d = .68). In addition, primary school males scored higher middle school females (t_(166)_ = 2.54, p = .05, d = .72, see Table 3). Other possible comparisons were nonsignificant (t_(166)_<.66, p>.05, d < .23). The "teacher” factor was nonsignificant (F_(3,166)_ = 1.69, p > .05, η_P_^2^ = .05, see Table 3).

**Table 3:** Shows the mean and standard deviation of each factor according to "school”, "sex”, and “teacher” factors (**∗-°^+#** highlighted the statistical difference in pairwise comparisons [p < .05]).

School	Sex	Teacher	Factor 1 (M±SD)	Factor 2 (M±SE)	Factor 3 (M±SE)	Factor 4 (M±SE)	Factor 5 (M±SE)	Factor 6 (M±SE)	Factor 7 (M±SE)
Primary			23.89 ± 1.84**∗**	12.46 ± 1.96	12.54 ± 2.11	7.92 ± 2.23	17.44 ± 2.60	12.71 ± 2.97	14.29 ± 1.23
Middle			22.38 ± 3.23**∗**	11.68 ± 2.04	12.01 ± 2.06	7.77 ± 2.29	17.58 ± 2.46	13.67 ± 1.77	14.32 ± 1.16
	Females		21.53 ± 3.74^**^**^	11.69 ± 2.08	11.75 ± 2.11	7.80 ± 2.26	17.45 ± 2.52	13.69 ± 1.85	14.31 ± .13
	Males		23.58 ± 1.89^**^**^	11.89 ± 2.01	12.40 ± 1.98	7.78 ± 2.30	17.47 ± 2.45	13.39 ± 2.14	14.31 ± .12
Primary	Females		24.11 ± 1.17^**°+**^	13.22 ± 1.64	12.78 ± 1.48	7.89 ± 1.97	17.22 ± 3.35	13.56 ± 2.19	14.44 ± 1.33
	Males		23.60 ± 2.17^**°**^	12.00 ± 2.04	12.40 ± 2.44	7.93 ± 2.30	17.80 ± 2.15	12.20 ± 3.32	14.20 ± 1.21
Middle	Females		21.22 ± 3.82^**°+**^	11.50 ± 2.06	11.62 ± 2.15**∗**	7.78 ± 2.44	17.47 ± 2.42	13.70 ± 1.82	14.29 ± 1.21
	Males		23.57 ± 1.85	11.86 ± 2.02	12.40 ± 1.88**∗**	7.75 ± 2.29	17.40 ± 2.52	13.64 ± 1.74;	14.30 ± 1.11
		Teacher 1	23.94 ± 1.93	12.81 ± 1.38; 12.0 ± 1.5^**-**^	12.63 ± 2.39	8.00 ± 2.44	17.63 ± 2.33	12.88 ± 3.62	14.75 ± 1.77
		Teacher 2	23.50 ± 1.84	11.75 ± 2.14	12.38 ± 2.03	7.75 ± 2.19	17.50 ± 2.80	12.38 ± 2.70	13.38 ± .45
		Teacher 3	22.28 ± 3.15	11.83 ± 2.10^**#**^	12.02 ± 1.76	7.67 ± 2.46	17.63 ± 2.33	13.71 ± 1.79	14.39 ± 1.06
		Teacher 4	22.59 ± 3.43	11.32 ± 1.87^**-#**^	11.98 ± 2.64	8.00 ± 1.82	16.98 ± 2.72	13.59 ± 1.76	14.14 ± 1.37

Artool linear regression analysis on Factor 2 indicated that "school” and "sex” factors were nonsignificant (F_(1,166)_ = .13, p > 05, η_P_^2^ = .00; F_(1,166)_ = .66, p > .05, η_P_^2^ = .00, respectively, see Table 3). The interaction “school” x “sex” was also nonsignificant (F_(1,166)_ = .72, p > .05, η_P_^2^ = .00, Table 3). The "teacher” factor was found to be significant (F_(3,166)_ = 2.69, p = .048, η_P_^2^ = .01, Table 3). Post-hoc analysis revealed a higher score for Teacher 1 (primary school) compared to Teacher 4 (middle school; t_(166)_ = 2.81, p = .03, d = .82). Other comparisons were nonsignificant (t(_166)<_2.02, p > .05, d < .64).

Artool linear regression analysis on Factor 3 revealed that "sex” and "school” factors were nonsignificant (F_(1,166)_ = 1.67, p > 05, η_P_^2^ = .01; F_(1,166)_ = 3.50, p > .05, η_P_^2^ = .02, respectively, Table 3). The interaction "school” x "sex” was also found to be nonsignificant (F_(1,166)_ = .16, p > .05, η_P_^2^ = .01, Table 3). The single factor "teacher” was nonsignificant (F_(3,166)_ = .72, p > .05, η_P_^2^ = .01, Table 3).

Artool linear regression analysis on Factor 4 highlighted that both "sex” and "school” were nonsignificant (F_(1,166)_ = .43, p > .05, η_P_^2^ = .00; F_(1,166)_ = .02, p > .05, η_P_^2^ = .00, respectively, Table 3). The interaction "sex” x "school” was also found to be nonsignificant (F_(1,166)_ = .09, p > .05, η_P_^2^ = .00, Table 3). The single factor "teacher” was nonsignificant (F_(3, 166)_ = .97, p > .05, η_P_^2^ = .00, see Table 3).

Artool analysis indicated on Factor 5 that both "sex” and "school” were found to be nonsignificant (F_(1,166)_ = .32, p > .05, η_P_^2^ = .00; F_(1,166)_ = .16, p > .05, η_P_^2^ = .00, respectively, Table 3). The interaction "sex” x "school” was also found to be nonsignificant (F_(1,166)_ = .25, p > .05, η_P_^2^ = .00, Table 3). The single factor "teacher” was also found to be nonsignificant (F_(3,166)_ = .54, p > .05, η_P_^2^ = .01, Table 3).

Artool linear regression analysis on Factor 6 highlighted that both "sex” and "school” factors and their interaction were nonsignificant (F_(1,166)_ = 3.01, p > .05, η_P_^2^ = .02; F_(1,166)_ = 1.46, p > .05, η_P_^2^ = .01; F_(1,166)_ = 3.32, p > .05, η_P_^2^ = .02, respectively). The "teacher” factor was also nonsignificant (F_(3,166)_ = .76, p > .05, η_P_^2^ = .01).

Artool analysis on Factor 7 showed that both "sex” and "school” single factors were nonsignificant (F_(1,166)_ = 1.51, p > .05, η_P_^2^ = .01; F(1,166) = .02, p > .05, η_P_^2^ = .00, respectively, Table 3). Whereas, the interaction "sex” x "school” was found to be significant (F_(1,166)_ = 5.44, p = .021, η_P_^2^ = .03). However, post-hoc analysis revealed no differences among the comparisons (t_(166)_<.85, p>.05, d < .35, Table 3). The "teacher” factor was nonsignificant (F_(3,166)_ = 1.56, p > .05, η_P_^2^ = .03, Table 3).

***Primary School Analysis*.** Artool regression analysis on Factor 1 revealed that both "sex” and "teacher” factors were nonsignificant (F_(1,22)_ = .12, p > .05, η_P_^2^ = .01; F_(1,22)_ = .96, p > .05, η_P_^2^ = .04, respectively, see Table 3).

Factor 2: Linear regression analysis found nonsignificance for the single factors "sex” and "teacher” (F_(1,20)_ = .00, p > .05, η_P_^2^ = .00; F_(1,20)_ = .04, p > .05, η_P_^2^ = .00, respectively, Table 3, Table 4).

**Table 4:** Shows the mean and standard deviationof each factor according to middle school classes and "sex” **(∗-°^** highlighted the statistical difference in pairwise comparisons [p < .05]).

School	Class	Sex	Factor 1 (M±SE)	Factor 2 (M±SE)	Factor 3 (M±SE)	Factor 4 (M±SE)	Factor 5 (M±SE)	Factor 6 (M±SE)	Factor 7 (M±SE)
Middle	I		23.21 ± 2.59	12.06 ± 2.05**∗**	12.43 ± 2.14; **∗**	8.45 ± 2.03**∗**	18.09 ± 2.05**∗**	8.45 ± 2.03	14.42 ± 1.02
	II		22.00 ± 3.38	10.89 ± 1.98**∗**^**-**^	11.51 ± 1.58; **∗**	7.38 ± 2.45	17.16 ± 2.35	7.38 ± 2.45	14.30 ± 1.39
	III		21.38 ± 3.72	11.76 ± 1.92^**-**^	11.76 ± 2.20;	7.02 ± 2.27**∗**	16.64 ± 2.90**∗**	7.02 ± 2.27	14.17 ± 1.17
	I	Females	22.60 ± 3.09**∗**	11.97 ± 2.02	12.27 ± 2.16;	8.24 ± 2.15	17.78 ± 2.28	8.24 ± 2.15	14.43 ± 1.04
		Males	23.97 ± 1.54^**-°**^	12.17 ± 2.12	12.63 ± 2.14;	8.70 ± 1.86	18.47 ± 1.70	8.70 ± 1.86	14.40 ± 1.00
	II	Females	20.14 ± 4.20^**°**^	10.21 ± 1.85	11.00 ± 1.36;	7.43 ± 2.77	17.50 ± 2.41	7.43 ± 2.77	14.29 ± 1.59
		Males	23.13 ± 2.18^**^**^	11.30 ± 1.99	11.83 ± 1.64;	7.35 ± 3.31	16.96 ± 2.35	7.35 ± 2.30	14.30 ± 1.30
	III	Females	19.65 ± 4.02^**+-**^**∗**^**^**^	11.52 ± 1.97	10.96 ± 2.29;	7.26 ± 2.18	16.96 ± 2.67	7.26 ± 2.18	14.09 ± 1.24
		Males	23.47 ± 1.81^**+**^	12.05 ± 1.87	12.74 ± 1.66;	6.74 ± 2.40	16.26 ± 3.20	6.74 ± 2.40	14.26 ± 1.10

Linear regression analysis on Factor 3 highlighted that both "sex” and "teacher” factors were found to be nonsignificant (F_(1,20)_ = .18, p > .05, η_P_^2^ = .01; F_(1,20)_ = .02, p > .05, η_P_^2^ = .00, respectively, Table 3, Table 4).

Artool linear regression on Factor 5 indicated that both "sex” and "teacher” single factors were nonsignificant (F_(1,22)_ = .09, p > .05, η_P_^2^ = .00; F_(1,22)_ = .06, p > .05, η_P_^2^ = .00, respectively, Table 3, Table 4).

Linear regression on Factor 5 revealed that both "sex” and "teacher” single factors were found to be nonsignificant (F_(1,22)_ = .00, p > .05, η_P_^2^ = .00; F_(1,22)_ = .06, p > .05, η_P_^2^ = .00, respectively, Table 3, Table 4).

Artool linear regression analysis on Factor 6 indicated that sex” and "teacher” factors were nonsignificant (F_(1,20)_ = .27, p > .05, η_P_^2^ = .01; F_(1,22)_ = .94, p > .05, η_P_^2^ = .04, respectively, Table 3, Table 4).

Artool linear regression analysis on Factor 7 showed that the "sex” factor was nonsignificant (F_(1,22)_ = .55, p > .05, η_P_^2^ = .02). The "teacher” factor was also nonsignificant (F_(1,22)_ = 3.42, p > .05, η_P_^2^ = .14, see Table 3, Table 4).

***Middle School Analysis*.** Artool liner regression analysis on Factor 1 indicated the significant effect of "class” on well-being scores (F_(2,140)_ = 7.79, p = .001, η_P_^2^ = .10). Post-hoc analysis revealed that a higher score for the first class compared to the second and third classes (t_(134)_ = 2.35, p = .05, d = .49; t_(134)_ = 3.76, p = .0006, d = .75). However, the differences between the second class and third class were nonsignificant (t_(136)_ = 1.14, p > .05, d = .26, respectively). "Sex” remained significant (F_(1,140)_ = 21.34 p < .0001, η_P_^2^ = .13). Male students reported a higher score than female students (Table 3). The interaction “class” x “sex” was significant (F_(2,140)_ = 5.36, p = .006, η_P_^2^ = .07, Table 4). Post-hoc analysis revealed that males of the third class had a higher score than their counterpart females (t_(140)_ = 3.84, p = .002, d = 1.09, Table 4). Moreover, first-class males reported a higher well-being score than third-class females (t_(140)_ = 5.52, p < .0001, d = 1.40). In addition, the first-class females reported higher well-being than third-class females (t_(140)_ = 3.93, p = .002, d = .96). Again, first-class males had a higher score than second-class females (t_(140)_ = 3.78, p = .003, d = 1.12) and second-class males had a higher score than third-class females (t_(140)_ = 3.73, p = .004, d = 1.01). The remaining comparisons were nonsignificant (t_(140)_<2.52, p>.05). The "teacher” factor was found to be nonsignificant (F_(1,144)_ = .49, p > .05, η_P_^2^ = .00).

Linear regression analysis on Factor 2 revealed that the single factor "class” was significant (F_(2,139)_ = 4.28, p = .02, η_P_^2^ = .06, Table 4). Post-hoc analysis revealed a higher score for the first-class compared to the second-class (t_(139)_ = 3.70, p = .009, d = .80). Higher score for the third-class compared to the second-class was found (t_(139)_ = 2.94, p = .01, d = .71). No differences between second- and third-class (t_(139)=_.44, p > .05, d = .08) were found. In addition, the analysis revealed that the single factor "sex” and its interaction with the "class” factor were nonsignificant (F_(1,139)_ = 2.42, p > .05, η_P_^2^ = .02; F(1,139) = .70, p > .05, η_P_^2^ = .01, respectively, Table 4). The "teacher” factor was found to be significant (F_(1,139)_ = 5.73, p = .018, η_P_^2^ = .04). Students reported a slightly higher score for Teacher 3 compared to Teacher 4 (see Table 3).

Artool linear regression on Factor 3 highlighted that the "class” factor was significant (F_(2,140)_ = 9.24, p = .002, η_P_^2^ = .06, Table 4). Post-hoc analysis revealed a higher score for the first-class compared to the second-class (t_(140)_ = 3.34, p = .003, d = .70). No differences emerged between the first- and third-class and between the second- and third-class (t(140) = 2.00, p > .05, d = .40; t(140) = 1.32, p > .05, d = .30). The "sex” factor was also significant (F_(1,140)_ = 5.94, p = .003, η_P_^2^ = .08) in which males had a higher score than females (see Table 3). The "teacher” factor, instead, was found to be nonsignificant (F_(1,144)_ = .41, p > .05, η_P_^2^ = .00, Table 3).

Artool linear regression analysis on Factor 4 revealed the single factor "class” was found to be significant (F_(2, 140)_ = 4.59, p = .01, η_P_^2^ = .06, Table 4). Post-hoc analysis revealed the score was higher for the first-compared to third-class (t_(140)_ = 2.82, p = .02, d = .55). No differences between the first- and second-class and between the second- and third-class were found (t_(140)_ = 2.07, p > .05, d = .43; t_(140)_ = .54, p > .05, d = .13, respectively).

The single factor "sex” and its interaction with the "class” factor were found to be nonsignificant (F_(1,140)_ = .52, p > .05, η_P_^2^ = .00; F_(1,140)_ = 1.47, p > .05, η_P_^2^ = .02, respectively).

The "teacher” factor was also found to be nonsignificant (F_(3,144)_ = .07, p > .05, η_P_^2^ = .00).

Artool linear regression analysis on Factor 5 showed that the "class” factor was significant (F_(2,140)_ = 3.73, p = .026, η_P_^2^ = .02). Post-hoc analysis revealed that the first-class had a higher score than the third-class (t_(140)_ = 2.48, p = .03, d = .49). No differences between the first- and second-class and between the second- and third-class (t_(140)_ = 1.97, p > .05, d = .41; t_(140)_ = .34, p > .05, d = .08, respectively). The "sex” factor (F_(1,140)_ = .23, p > .05, η_P_^2^ = .00) and its interaction with the "class” factor (F_(2,140)_ = .17, p > .05, η_P_^2^ = .03) were found to be nonsignificant. The "teacher” factor was found to be nonsignificant (F_(1,144)_ = 1.80, p > .05, η_P_^2^ = .01).

Artool linear regression on Factor 6 highlighted that both "sex” and "class” factors and their interaction were nonsignificant (F_(1,140)_ = 1.63, p > .05, η_P_^2^ = .02; F_(1,140)_ = .27, p > .05, η_P_^2^ = .00; F_(1,140)_ = .26, p > .05, η_P_^2^ = .01, respectively). The "teacher” factor was also nonsignificant (F_(1,144)_ = .38, p > .05, η_P_^2^ = .00).

Artool linear regression on Factor 7 indicated that "class” and "sex” factors and their interaction were nonsignificant (F_(1,140)_ = 1.21, p > .05, η_P_^2^ = .02; F_(1,140)_ = .60, p > .05, η_P_^2^ = .00; F_(1,140)_ = .72, p > .05, η_P_^2^ = .01, respectively). The "teacher” factor was also found to be nonsignificant (F_(1,140)_ = .71, p > .05, η_P_^2^ = .01).

## 4 Discussion

This study investigated and explored PE class experiences and perceptions from the point of view of primary and middle school students. We found higher well-being and enjoyment related to PE classes in primary school students than in middle school students. In addition, we found higher well-being and enjoyment in males than in females.

In this study, we focused on different social aspects on collaboration, well-being, teachers' behavior, inclusion, and general behavior. A questionnaire was employed, and the EFA identified seven factors: well-being (Factor 1), physical activities for all students (Factor 2), teacher-proposed activity difficulty/plainness (Factor 3), teacher-oriented behavior to results (Factor 4), perception of activities with disabled students and collaboration (Factor 5), physical activities orientation (Factor 6), and attention towards nondisabled and disabled students (Factor 7). We also controlled the potential impacts of class (age) and sex.

Results indicated higher well-being and enjoyment perception (e.g., enjoyment in participation in PE classes and staying together with classmates) for primary school students compared to middle school students and for younger compared to older students in middle schools. Furthermore, males reported a higher well-being than females. Possible reasons for this finding include the greater amount of time primary school teachers spend with their students, allowing for more personalized attention and support, and the generally more inclusive and collaborative nature of primary school environments. Additionally, younger students may naturally have higher levels of well-being due to less academic pressure and more opportunities for play and physical activity.

Regarding activities designed for everyone, no differences were found between the two school types, but younger middle school students perceived better adaptation than older students. Differences between Teacher 1 and Teacher 4 and between Teacher 3 and Teacher 4 were also found. Teacher 4 had a relatively lower score compared to Teachers 1 and 3 (11.32 ± 1.87 out of 15 points). However, the perception of these items can vary across ages and abilities; thus, these results may be spurious and are not directly imputable to the Teacher. In other words, the teacher is/was genuinely trying to create activities for everyone, but it may be misperceived by students. In future research, it seems very important to control this factor because the role of the teacher is vital in creating enjoyable and inclusive PE classes.

Age effects were evident in the teacher's oriented behavior to results and activities with disabilities students and collaboration. Younger middle school students reported teachers paying more attention to disabled students, though a decline was noted over the years. This might be due to increased academic pressures in higher grades, which can limit the time and resources teachers have to focus on inclusivity. These age effects imply that younger students benefit more from inclusive practices, highlighting the need for sustained efforts to maintain these practices throughout the school curriculum. On the other hand, no differences according to sex, age, and school grade were found in PE orientation and attention toward nondisabled and disabled students (Factor 7). These results confirm that, to some extent, teachers recognized the significance of directing their attention toward students to create a positive and inclusive environment.

A previous systematic review [34] on inclusive PE highlighted that, from a philosophical standpoint, all teachers acknowledge the importance of cultivating inclusive PE classes. However, in practice, teachers encountered challenges in planning their actions.

Our results also indicated sex differences in well-being perception in both primary and middle school. These findings align with previous investigations (e.g., 4,25,33). In addition, Johnson and colleagues [35] reported higher enjoyment among males compared to females during a PE intervention, suggesting a persistent cultural issue. Generally, females appear to exhibit a lower inclination toward physical or sport activities, a trend reflected in disparities in physical activity and fitness levels between genders [36,37]. Consequently, teachers and parents should promote the participation of female students in physical activities, and teachers and coaches should ensure equal opportunities for both sexes/genders in all sports and physical education programs. Additionally, implementing policies that mandate equal access to sports facilities and resources can further support gender equality in physical education.

Our findings confirm that in primary school, teachers seem to be more sensitive to the well-being of the students. This observation could be attributed, in part, to the substantial amount of time primary school teachers spend with their students, which far exceeds the time allocated by PE teachers in middle school (i.e., 2 h per week). Thus, the promotion of methods such as the “school on the move” [38,39] where students can learn math, literacy, or other subjects using PE should be encouraged. At the same time, these methods seem to be able to increase the physical activity levels and improve the knowledge of the specific subject.

However, the prevailing body of research indicates that the incorporation of students with disabilities is not yet widely practiced [40]. Thus, to promote inclusion, teachers can engage disabled and nondisabled children using reverse inclusion or simulation [41,42] strategies. In particular, employing activities such as Integrated Basketball, Wheelchair basketball, Goalball, or Sitting volley [[43], [44], [45]] can reduce inequities between disabled and nondisabled students. Our results also highlighted higher well-being and enjoyment in PE classes for males compared to females; thus, to reduce the inequities between the sexes, teachers can instead develop activities for both sexes while parents or legal guardians can be encouraged to support and motivate their daughters to engage in physical and sport-related pursuits.

In general, PE teachers, teachers, and educators should receive specific training to enhance their ability to create inclusive and enjoyable PE classes. This training should include strategies for adapting activities to meet diverse student needs and promoting collaboration among all students. On the other hand, policymakers and schools should support initiatives that promote inclusive practices in PE, such as providing resources for adapted equipment and training programs for teachers.

### 4.1 Limitations

We used an ad-hoc questionnaire to evaluate students' perceptions of PE classes. The EFA results demonstrated a commendable level of consistency among the questionnaire items analyzed. However, some Cronbach's alphas were at the limit of acceptance (e.g., Factor 2 = .41). Another possible limitation includes the small sample size of primary school students and the modest effect size in middle school data analysis.

Regrettably, the recruitment of students remains challenging, even with government prioritization of youth health promotion, as some schools and teachers perceive these endeavors as unproductive or time-wasting [46,47].

Another potential limitation is that the responses are based on individual perceptions, which may be influenced by personal biases or the desire to provide socially desirable answers, especially to the questions related to teachers’ behavior.

Future research will involve increasing the sample size of primary school students and refining the questionnaire by eliminating or modifying specific questions to align with the study's objectives. Additionally, a Confirmatory Factor Analysis will be conducted to scrutinize the potential validity of the questionnaire.

Another limit is the absence of qualitative or quantitative input from students with disabilities hinders insight into overall inclusivity during PE classes.

In future research, obtaining the perceptions of disabled students through both quantitative and qualitative tools will be essential for informing the adaptation or reform of physical education programs to cater to diverse student needs.

## 5 Conclusion

This study furnishes insights into students' perceptions across various dimensions of PE classes. Younger students appear to exhibit a higher level of well-being than their older counterparts. This discrepancy may stem from distinct approaches employed in primary and middle schools or could be attributed to maturation factors. From an inclusion standpoint, it is noteworthy that students across all grades reported a consistently high level of inclusion. This means that teachers are aware of the importance of creating inclusive lessons.

Finally, findings underscore the importance of tailored PE programs that consider the individual needs of the student, to behave differently according to student age and sex/gender to enhance the overall well-being and inclusivity of PE classes. Implementing such programs can help ensure that all students, regardless of their background or abilities, can participate fully and benefit from physical education.

## CRediT authorship contribution statement

**Gabriele Russo:** Writing – original draft, Methodology, Investigation, Formal analysis, Data curation, Conceptualization. **Alice Masini:** Writing – review & editing, Supervision. **Laura Dallolio:** Supervision, Methodology. **Andrea Ceciliani:** Writing – review & editing, Supervision, Project administration, Methodology, Conceptualization.

## Data availability statement

The dataset is available at the following link: https://osf.io/vwtkb/?view_only=6f26bd1e1ac24d39831ca6cd103d0194.

## Declaration of competing interest

The authors declare that they have no known competing financial interests or personal relationships that could have appeared to influence the work reported in this paper.
