# Supplementary material for: Primary and middle school students' views on inclusive physical education: Perceptions, practices, and future directions
Source: Heliyon. 2024 Dec 14;11(1):e41232. doi: 10.1016/j.heliyon.2024.e41232 (PMC11719291; doi:10.1016/j.heliyon.2024.e41232)
Supplement: Multimedia component 1 [file mmc1.docx]

Table 2 shows all questionnaire items. Each participant responded on a Likert-5 scale from "not at all" to "a lot."

| Item | Question | Factor | Item | Question | Factor |
| --- | --- | --- | --- | --- | --- |
| 1 | All students are involved in Physical Education lessons | 2 | 16 | The teacher willingly works with the whole class | 7 |
| 2 | When someone is in difficulty, they can rely on the help of their classmates |  | 17 | I enjoy activities with students with disabilities | 5 |
| 3 | During the physical education class, the teacher is attentive to students with difficulties | 7 | 18 | I feel sorry for students with disabilities |  |
| 4 | When someone is in difficulty, they can count on the help of the Physical Education teacher |  | 19 | Games suitable for students with disabilities are boring | 5 |
| 5 | I willingly participate in Physical Education classes | 1 | 20 | During Physical Education classes, we also work in pairs |  |
| 6 | I have fun during Physical Education classes | 1 | 21 | During Physical Education classes, we also work in small groups |  |
| 7 | During the Physical Education class, games and activities involve everyone | 2 | 22 | If someone is in difficulty, the teacher helps them | 5 |
| 8 | Games and activities are accessible to everyone | 3 | 23 | During Physical Education classes, we feel good together | 1 |
| 9 | The games are achievable by everyone | 3 | 24 | The teacher is attentive to the effort | 5 |
| 10 | Students with disabilities are treated well | 7 | 25 | The teacher is attentive to the result |  |
| 11 | Students with disabilities are facilitated too much |  | 26 | The teacher always praises those with the best results in activities | 4 |
| 12 | Games and activities involve everyone | 2 | 27 | The teacher praises the most skilled | 4 |
| 13 | During Physical Education classes, we work a lot together | 3 | 28 | The teacher is attentive to those with worse results |  |
| 14 | The teacher proposes activities suitable for the most skilled | 6 | 29 | The teacher only addresses the most skilled | 6 |
| 15 | The teacher only rewards the best | 6 | 30 | During Physical Education classes, I feel good | 1 |
|  |  |  | 31 | During Physical Education hours, I feel at ease | 1 |
