# Supplementary material for: Primary and middle school students' views on inclusive physical education: Perceptions, practices, and future directions
Source: Heliyon. 2024 Dec 14;11(1):e41232. doi: 10.1016/j.heliyon.2024.e41232 (PMC11719291; doi:10.1016/j.heliyon.2024.e41232)
Supplement: Multimedia component 2 [file mmc2.docx]

Table S1 shows the Spearman correlation matrix among all questionnaire items.

| Item | | 1 | 2 | 3 | 4 | 5 | 6 | 7 | 8 | 9 | 10 | 11 | 12 | 13 | 14 | 15 | 16 | 17 | 18 | 19 | 20 | 21 | 22 | 23 | 24 | 25 | 26 | 27 | 28 | 29 | 30 | 31 |
| --- | --- | --- | --- | --- | --- | --- | --- | --- | --- | --- | --- | --- | --- | --- | --- | --- | --- | --- | --- | --- | --- | --- | --- | --- | --- | --- | --- | --- | --- | --- | --- | --- |
| 1 | | 1 | .30 | .10 | .17 | .12 | .08 | .46 | .28 | .25 | .09 | .14 | .47 | .31 | .14 | .06 | .07 | -.06 | .09 | .09 | -.13 | .11 | .15 | .26 | .06 | .06 | .12 | .08 | -.11 | .01 | .25 | .20 |
| 2 | |  | 1 | .25 | .34 | .30 | .35 | .31 | .19 | .25 | .28 | .05 | .29 | .34 | .26 | .20 | .24 | .17 | .04 | .24 | .06 | .16 | .35 | .38 | .17 | -.05 | .18 | .22 | -.02 | .12 | .31 | .28 |
| 3 |  |  | 1 | .28 | .21 | .07 | .20 | .19 | .13 | .32 | .05 | .13 | .15 | .15 | .02 | .32 | .11 | .09 | .16 | .18 | -.04 | .19 | .09 | .25 | -.11 | .03 | -.05 | .01 | .02 | .10 | .02 |  |
| 4 |  |  |  | 1 | .02 | .01 | .21 | .23 | .28 | .27 | .04 | .24 | .23 | .11 | .14 | .44 | .20 | -.03 | .25 | .16 | .17 | .41 | .01 | .38 | -.12 | .08 | .07 | .04 | .22 | -.04 | .00 |  |
| 5 |  |  |  |  | 1 | .57 | .15 | .24 | .20 | .25 | .08 | .19 | .27 | .16 | .09 | .12 | .03 | .10 | .09 | -.04 | -.08 | .18 | .46 | .09 | -.05 | .12 | .16 | -.10 | .08 | .55 | .44 |  |
| 6 |  |  |  |  |  | 1 | .13 | .13 | .09 | .15 | -.07 | .18 | .31 | .11 | .08 | .17 | .09 | .02 | .01 | -.07 | -.03 | .11 | .52 | -.04 | -.14 | -.01 | .17 | .01 | .03 | .61 | .46 |  |
| 7 |  |  |  |  |  |  | 1 | .41 | .27 | .26 | .12 | .69 | .36 | .13 | .03 | .22 | .04 | .03 | .19 | -.09 | .08 | .28 | .24 | .15 | .08 | .20 | .17 | -.07 | .24 | .20 | .16 |  |
| 8 |  |  |  |  |  |  |  | 1 | .47 | .22 | .10 | .40 | .35 | .11 | .00 | .32 | .10 | .13 | .16 | -.05 | .12 | .25 | .14 | .11 | -.02 | .18 | .20 | -.02 | .07 | .13 | .18 |  |
| 9 |  |  |  |  |  |  |  |  | 1 | .16 | .12 | .47 | .37 | .03 | .03 | .23 | .11 | .01 | .10 | .01 | .13 | .25 | .20 | .29 | -.06 | .04 | .02 | .00 | -.04 | .17 | .10 |  |
| 10 |  |  |  |  |  |  |  |  |  | 1 | .01 | .26 | .18 | .22 | .04 | .36 | .27 | .15 | .34 | -.02 | .03 | .33 | .21 | .20 | .00 | .07 | .09 | .03 | .14 | .15 | .02 |  |
| 11 |  |  |  |  |  |  |  |  |  |  | 1 | .07 | .07 | .17 | .04 | -.01 | .05 | .24 | .07 | .09 | .05 | .02 | .11 | .06 | .08 | .18 | .12 | .05 | .10 | .05 | .14 |  |
| 12 |  |  |  |  |  |  |  |  |  |  |  | 1 | .48 | .19 | .14 | .25 | .07 | .00 | .11 | -.10 | .08 | .26 | .31 | .23 | -.04 | .14 | .09 | -.05 | .10 | .24 | .15 |  |
| 13 |  |  |  |  |  |  |  |  |  |  |  |  | 1 | .09 | .09 | .26 | .13 | .00 | .07 | .01 | .11 | .24 | .45 | .29 | -.05 | .10 | .13 | .03 | .06 | .40 | .37 |  |
| 14 |  |  |  |  |  |  |  |  |  |  |  |  |  | 1 | .42 | .19 | -.08 | .20 | .16 | -.04 | .03 | .09 | .21 | -.02 | .09 | .32 | .34 | -.10 | .24 | .19 | .08 |  |
| 15 |  |  |  |  |  |  |  |  |  |  |  |  |  |  | 1 | .18 | .01 | .08 | .13 | -.02 | .06 | .09 | .05 | .10 | .06 | .27 | .31 | -.11 | .41 | .18 | .04 |  |
| 16 |  |  |  |  |  |  |  |  |  |  |  |  |  |  |  | 1 | .25 | -.04 | .30 | .02 | .12 | .35 | .10 | .24 | -.03 | .16 | .15 | -.01 | .28 | .06 | .03 |  |
| 17 |  |  |  |  |  |  |  |  |  |  |  |  |  |  |  |  | 1 | .10 | .45 | -.03 | .03 | .29 | .08 | .25 | -.04 | -.01 | .07 | .10 | .14 | -.01 | -.03 |  |
| 18 |  |  |  |  |  |  |  |  |  |  |  |  |  |  |  |  |  | 1 | .38 | .00 | .08 | -.07 | .11 | .05 | -.05 | .06 | .10 | .08 | .19 | .00 | .11 |  |
| 19 |  |  |  |  |  |  |  |  |  |  |  |  |  |  |  |  |  |  | 1 | .08 | .14 | .29 | .10 | .22 | -.04 | .12 | .20 | -.03 | .23 | .00 | .06 |  |
| 20 |  |  |  |  |  |  |  |  |  |  |  |  |  |  |  |  |  |  |  | 1 | .21 | .08 | -.08 | .17 | -.02 | -.01 | .06 | .08 | .01 | -.10 | -.03 |  |
| 21 |  |  |  |  |  |  |  |  |  |  |  |  |  |  |  |  |  |  |  |  | 1 | .19 | .11 | .18 | -.13 | -.06 | -.06 | .14 | -.04 | -.04 | .00 |  |
| 22 |  |  |  |  |  |  |  |  |  |  |  |  |  |  |  |  |  |  |  |  |  | 1 | .18 | .47 | -.04 | -.03 | .02 | -.01 | .14 | .17 | .08 |  |
| 23 |  |  |  |  |  |  |  |  |  |  |  |  |  |  |  |  |  |  |  |  |  |  | 1 | .06 | .03 | .07 | .22 | -.03 | -.03 | .58 | .48 |  |
| 24 |  |  |  |  |  |  |  |  |  |  |  |  |  |  |  |  |  |  |  |  |  |  |  | 1 | -.16 | -.09 | -.06 | .10 | .10 | .07 | .03 |  |
| 25 |  |  |  |  |  |  |  |  |  |  |  |  |  |  |  |  |  |  |  |  |  |  |  |  | 1 | .32 | .28 | -.27 | -.01 | -.06 | .07 |  |
| 26 |  |  |  |  |  |  |  |  |  |  |  |  |  |  |  |  |  |  |  |  |  |  |  |  |  | 1 | .69 | -.25 | .33 | .09 | .06 |  |
| 27 |  |  |  |  |  |  |  |  |  |  |  |  |  |  |  |  |  |  |  |  |  |  |  |  |  |  | 1 | -.27 | .34 | .17 | .12 |  |
| 28 |  |  |  |  |  |  |  |  |  |  |  |  |  |  |  |  |  |  |  |  |  |  |  |  |  |  |  | 1 | -.11 | -.10 | .03 |  |
| 29 |  |  |  |  |  |  |  |  |  |  |  |  |  |  |  |  |  |  |  |  |  |  |  |  |  |  |  |  | 1 | .00 | -.02 |  |
| 30 |  |  |  |  |  |  |  |  |  |  |  |  |  |  |  |  |  |  |  |  |  |  |  |  |  |  |  |  |  | 1 | .63 |  |
